# Hydrophobic UV-Curable Powder Clear Coatings: Study on the Synthesis of New Crosslinking Agents Based on Raw Materials Derived from Renewable Sources

**DOI:** 10.3390/ma14164710

**Published:** 2021-08-20

**Authors:** Dominika Czachor-Jadacka, Barbara Pilch-Pitera, Maciej Kisiel, Justyna Gumieniak

**Affiliations:** 1 Faculty of Chemistry, Department of Polymers and Biopolymers, Rzeszow University of Technology, ul. Powstańców Warszawy 6, 35-959 Rzeszów, Poland; d440@stud.prz.edu.pl; 2 Faculty of Chemistry, Department of Industrial and Materials Chemistry, Rzeszow University of Technology, ul. Powstańców Warszawy 6, 35-959 Rzeszów, Poland; m.kisiel@prz.edu.pl; 3 Faculty of Mechanics and Technology, Rzeszow University of Technology, ul. Kwiatkowskiego 4, 37-450 Stalowa Wola, Poland; j.gumieniak@prz.edu.pl

**Keywords:** UV-curable powder coatings, crosslinking agent, urethane acrylates, xylitol, DMA

## Abstract

Methods for the synthesis of urethane acrylates used as new crosslinking agents for hydrophobic UV-curable powder clear coatings were developed. In the synthesis of urethane acrylates, isophorone diisocyanate, glycerin, xylitol, polyethylene glycol and polysiloxane KF-6000, as well as 2-hydroxyethyl acrylate or 2-hydroxyethyl methacrylate, were used. In order to increase the functionality of urethane acrylates, glycerin and xylitol derived from renewable sources were introduced. The chemical structure of the urethane acrylates was verified by IR spectroscopy. UV-curable powder clear coatings were obtained through a combination of urethane acrylates with unsaturated polyester resins. The thermal behavior and crosslinking density were examined using DMA. The obtained coatings were evaluated by performing the following tests: roughness, gloss, scratch resistance, hardness, adhesion to steel and water contact angle. As part of this research, high hydrophobicity and scratch resistance of UV-curable powder clear coatings were developed, which are a VOC-free and economically attractive alternative method for low thermal resistance surface protection, such as for composites, wood and wood-based materials.

## 1. Introduction

Recent years have shown a growing interest in ecological, environmentally friendly coatings which will still offer various functionalities, and thus meet consumers’ requirements in furniture, civil engineering and anti-corrosion applications. Powder coatings are among the products that are environmentally friendly. They are characterized by a very low level of VOC (Volatile Organic Compound) emissions, high product performance and low waste generation. Research has shown that VOC emissions from powder coatings are in the range of 0.4–3.0% depending on the chemical composition of the resin [1]. Such a low VOC emission of powder coatings is due to the lack of a liquid vehicle, compared to conventional liquid paints. In the case of liquid paints, most often toxic organic solvents or water are used as the vehicle. In solvent-borne products, compounds composed of 6–10 carbon atoms are mainly emitted, such as toluene, ethylbenzene and xylene [2]. In the case of solvent-based products, VOC emission can reach up to 83.0%, while in water-based products it is much lower, e.g., in latex paints it is 6.6% [3]. In latex paints, the main emission source can be toxic formaldehyde, which is added as an in-can biocide, as well as other additives such as ammonia and coalescent agents, e.g., texanol. Powder coatings are mostly used only for coating substrates resistant to high temperatures, i.e., metal, ceramics or glass. High temperatures in the range of 160–200 °C for a period 5–20 min must be ensured during their curing. However, with the passing of time, changes and requirements in this area, a lot of research has been carried out on powder coatings for heat-sensitive substrates such as MDF (Medium-density fibreboard), wood panels or plastic [3,4,5,6,7]. For this purpose, one possibility is to use UV light. Due to advantages such as high curing speed and good mechanical properties of the crosslinked coatings, ultraviolet (UV) curing has become a fast and environmentally friendly curing technique [7,8,9,10].

SMC Corporation developed the first UV-curable powder coating based on acrylated epoxy and acrylated polyester in 1978. At the time, this formulation offered relatively good, state of the art properties and provided no orange peel effect [11]. Research on UV-curable coatings has developed at an increasingly rapid rate. Various types of resins have emerged, including urethane acrylates (UAs), unsaturated polyester resins [12,13,14,15], (meth)acrylic resins [16,17], epoxy resins [18,19], polyamides [20,21], poly (limonene carbonates) and hyperbranched polymers [3,5,21,22,23,24].

Wenning presented urethane acrylates as a new type of resin for producing light-induced transparent powder coatings. A combination of amorphous and three (semi) crystalline urethane acrylates was selected as the binder. By mixing them in appropriate proportions, the glass transition temperature and viscosity can be controlled [25]. In a subsequent study in 2003, Wenning et al. described a radiation-curable powder composition containing amorphous urethane acrylate, which was obtained by reacting a polyisocyanate (IPDI (isophorone diisocyanate) is preferred) with a compound containing OH groups and a polymerizable acrylate group (hydroxyethyl acrylate is preferred). The coatings were light, weather stable, flexible, hard and had good adhesion to steel panels [26]. Maurin et al. described a study on the incorporation of silver into a UV powder formulation to develop an antimicrobial coating for wood-based panels. Urethane diacrylate was also chosen as the UV-curable powder resin for the study. UA is an amorphous solid and has a glass transition temperature (Tg) close to 56 °C and the molar mass (Mn) was estimated to be 2000 g/mol. The obtained UV-curable powder coatings could be used as protective and decorative coatings for wood [27].

UV-curable powder coatings give the possibility to replace liquid lacquers in the furniture industry for coating wood or MDF. In addition, a hydrophobic coating ensures better protection of the substrate from moisture and dirt. Moreover, using natural ingredients results in less environmental pollution because they come from renewable sources whose production has less impact on the environment. Li et al. used limonene derivatives for the synthesis of biobased epoxides and obtained UV-curable coatings [28].

The main objective of this study was to develop a method for the synthesis of new urethane acrylates as crosslinkers for hydrophobic UV-curable powder clear coatings for wood-based materials such as MDF or wood. The increase in functionality of polyisocyanates (PICs) was obtained by using a mixture of glycerol and xylitol for their synthesis, and a more hydrophobic characteristic was achieved by introducing a hydroxyl terminated polysiloxane into their structure. Moreover, 2-hydroxyethyl acrylate and 2-hydroxyethyl methacrylate were used as the components containing unsaturated bonds that polymerize during crosslinking under UV light.

## 2. Experimental

### 2.1. Raw Materials and Reagents

Materials included isophorone diisocyanate (IPDI) from Evonic Industries (Essen, Germany), dibutyltin dilaurate (DTBL) from Sigma-Aldrich (Buchs, Switzerland) glycerin from Chempur (Piekary Śląskie, Poland), polyethylene glycol (Mw = 300 g/mol) from POCH-Gliwice S.A. (Gliwice, Poland), α,ω-(hydroxyethyleneoxypropylene) polydimethylsiloxane (KF-6000), hydroxyl value: 120 mg KOH/g from Shin-Etsu (Tokyo, Japan), xylitol from J&K Scientific (Beijing, China), 2-hydroxyethyl acrylate (HEA) and 2-hydroxyethyl methacrylate (HEMA) from Merck (Darmstadt, Germany) and hydroquinone from Alfa Aesar (Haverhill, MA, USA). For powder coatings, hydroxyl terminated unsaturated polyester resin (hydroxyl value: 54.7 mg KOH/g, η_165_ = 6390 Pa⋅s, Tg = 75 °C (DMA)) from Ciech (Nowa Sarzyna, Poland), photoinitiator Irgacure 651, flow control agent Resiflow PV 88 supplied by Worlèe Chemie GmbH (Lauenburg, Germany) and benzoin as degassing agent purchased from Sigma-Aldrich (Buchs, Switzerland) were used.

### 2.2. A Method for the Synthesis of Crosslinking Agents

The synthesis can be divided into two steps: a polyisocyanate (PIC) was produced in the first step and acrylate or methacrylate containing hydroxyl groups reacted with the isocyanate groups of the polyisocyanate in the second step. Isophorone diisocyanate (IPDI) and dibutyltin dilaurate as a catalyst (0.1 wt% with respect to diisocyanate) were placed in a glass reactor fitted with a reflux condenser, temperature control, steel agitator, nitrogen inlet and dropping funnel. Meanwhile, a mixture of hydroxyl component, glycerin and xylitol, was made. A beaker with the mixture was placed on a magnetic stirrer at 80 °C and 900 rpm to dissolve the sugar alcohol. After dissolving the xylitol, polyethylene glycol or KF-6000 was then added to the mixture. The mixture was then introduced dropwise into the diisocyanate in the glass reactor. The reaction was carried out at 95 °C for two hours. Then, the reaction mixture was cooled to 80 °C. A calculated amount of 2-hydroxyethyl acrylate or 2-hydroxyethyl methacrylate with hydroquinone (0.1 wt% with respect to 2-hydroxyethyl acrylate or 2-hydroxyethyl methacrylate) was added to the reaction mixture in each case (the molar ratio of -NCO group to -OH at 1:1). The reaction progress was monitored with FT-IR. After the absorption band derived from the -NCO group in the FT-IR spectrum at 2264 cm^−1^ had completely disappeared, the reaction was terminated. The crosslinking agents were named after the first letters of the names of the substrates, e.g., IGKFX/HEA means a PIC made from IPDI, glycerin, KF-6000, xylitol combined with 2-hydroxyethyl acrylate; IGKFX/HEMA means a PIC made of IPDI, glycerin, KF-6000, xylitol combined with 2-hydroxyethyl methacrylate.

### 2.3. Preparation of Powder Compositions and Coatings

The UV-curable powder clear coating compositions consisted of the crosslinking agent, unsaturated polyester resin, photoinitiator Irgacure 651 (2.5 wt%), degassing agent (0.8 wt%) and leveling agent (1 wt%). The prepared mixture was milled and extruded in a co-rotating twin screw mini extruder EHP 2 × 12 Sline from Zamak (Skawina, Poland). Temperature distribution in the extruder was as follows: zone I—95 °C, zone II—102 °C, zone III—107 °C, adapter—115 °C. Screw rotational speed was 125 rpm. The next step after the extrusion process was to cool the mixture, powder it and then sieve it through a 100 μm sieve. The final powder coatings were treated with a PEM X-1 electrostatic gun, which was controlled with an EPG Sprint X (CORONA) device made by Wagner (Altstatten, Switzerland). Powder coatings were applied to previously prepared steel panels, MDF and wood panels. The applied electrode voltage was 30 kV. In this gun-spraying method, powder particles were transported from the tank by compressed air and electrified by an electrode placed in the gun nozzle. The charged particles were then transferred to grounded steel plates, MDF and wood panels. To prepare the steel plates, they first needed to be degreased with acetone and then immersed in a 1.5% aqueous solution of ESKAPHOR Z 2000C with pH = 5.5 for 4 min to apply the conversion phosphate–zirconium coating. After being removed from the solution, the plates were then washed with distilled water and dried. The MDF panel was cleaned with sandpaper and then dried in an oven to obtain a moisture content in the range of 4–8%. The following step was the curing stage. First, the coatings were melted in an oven at 110 °C for 5 min and then cured using a Dymax UVC-5 Compact Light-Curing Conveyor System equipped with a mercury lamp (high-power lamp 850 W). The obtained coatings were named after the PIC used as the crosslinking agent, e.g., L-IGKFX/HEMA means a coating cured with IGKFX/HEMA.

## 3. Measurements

### 3.1. Characterization of Crosslinking Agents

#### FT-IR Structural Analysis

The Thermo Scientific Nicolet 6700 FT-IR (Thermo Fisher Scientific, Waltham, MA, USA) spectrophotometer was applied for recording of crosslinking agents’ IR spectra.

### 3.2. Characterizations of Cured Coatings

#### 3.2.1. DMA Measurements

The DMA/SDTA861e unit from Mettler Toledo (Mettler-Toledo, Columbus, OH, USA) was used for analysis. The analysis conditions were as follows:

- tension mode at a constant frequency: 1 Hz,
- heating rate: 3 °C/min,
- displacement amplitude: max. 10 μm,
- force amplitude: max. 0.1 N,
- temperature range: 0–200 °C,
- sample dimensions: 0.15 × 6.50 × 5.50 mm.

#### 3.2.2. XPS Analysis

A K-Alpha™ X-ray Photoelectron Spectrometer (XPS) from Thermo Scientific™ (Waltham, MA, USA) was used for analysis. The analysis conditions were as follows:

- X-ray source: monochromatic Al K-alpha,
- energy of X-ray source: 1486.68 eV,
- power of X-ray source: 360 W,
- vacuum range: 10^−9^–10^−8^ mbar,
- sample dimensions: 1 × 1 cm,
- sample substrate: steel.

#### 3.2.3. Polymerization Test

The test was performed according to the Qualicoat technical requirements [29]. A cotton wool swab was soaked in methyl ethyl ketone (MEK). The coatings were rubbed back and forth with a soaked cotton swab 30 times. Then, 30 min after rubbing, the coating was assessed on a four-point scale. The best (fourth) degree means no noticeable change. Degree 3 is assigned when the coating has a gloss loss of less than 5 units. If the coating becomes matte and it is possible to scratch it with a fingernail, it is classified as the second degree of damage. The first degree is assigned when the coating is deep matte and soft.

#### 3.2.4. Roughness

A Mar Surf PSI profilometer (Mahr GmbH, Göttingen, Germany) was used to measure coating roughness according to standard PN-EN ISO 12085.

Measurements of the surface roughness of the coatings were carried out with a Mahr GmbH Göttingen profilometer (Mahr GmbH, Göttingen, Germany) of the Mar Surf PS1 type according to standard PN-EN ISO 12085. Measurements were taken in different parts of the sample with a measuring length equal to LT = 5.600 mm. The measurement results were presented in the form of roughness parameters Ra and Rz.

#### 3.2.5. Gloss

The coating gloss was measured by means of a micro-TRI-gloss µ gloss meter from Byk-Gardner (Geretsried, Germany) according to standard PN-EN ISO 2813.

#### 3.2.6. Adhesion to Steel

The cross-cut test was performed according to standard PN-EN ISO 2409. Six cutters knife from Byk-Gardner (Geretsried, Germany) were used in order to make an incision through the coating into the substrate. The spacing between of cutters was 2 mm.

The incisions were classified on a six-degree scale from 0 to 5. Degree 0 means the best adhesion to the substrate, while degree 5 means the worst adhesion.

#### 3.2.7. Hardness

A König Pendulum tester from Byk-Gardner (Geretsried, Germany) was used to determine the hardness of the coatings, in accordance with standard PN-EN ISO 1522. Relative hardness was calculated as the quotient of the pendulum oscillation time on the investigated coating to pendulum oscillation time on glass.

#### 3.2.8. Scratch Resistance

The scratch resistance test was carried out using the Clemen tester (Elcometer, Manchester, UK) according to PN-EN ISO 1518-1. The measurement consisted in determining the smallest load at which the coating was scratched with a semicircular tip needle.

#### 3.2.9. Water Contact Angle

The water contact angle of the coatings was determined by the “sitting drop” method with the use of an OCA15 EC optical goniometer by Data Physics according to standard EN 828:2000. The volume of the measuring drop was 1 μL and the measurement temperature was 24 ± 1 °C. The drop images were recorded with a camera. The contact angle was determined by means of a control program, after prior determination of the baseline and the drop contour. The arithmetic mean of 10 determinations was adopted as the final result.

## 4. Results and Discussion

Cycloaliphatic isocyanate (IPDI) was used to obtain the PIC, because the produced coatings have a reduced tendency for yellowing. Additionally, coatings containing IPDI typically exhibit excellent light and weather stability and are usually used for outdoor applications. Xylitol, which was readily soluble in glycerol, was chosen to branch the polyisocyanate structure. By using glycerin as a solvent, another solvent, such as acetone, MEK or DMSO, could be eliminated. Due to the high viscosity of urethane acrylate, these solvents would be challenging to vaporize. In this study, glycerin was attached to IPDI to produce polyisocyanate. Xylitol, which is a readily available ingredient from renewable sources, was also selected as the polyalcohol. Polyethylene glycol with Mw = 300 g/mol ensures adequate flexibility of the PIC. Polysiloxane KF-6000 was also incorporated into the PIC structure to make the coating more flexible and additionally to make the coating hydrophobic. The used polysiloxane had the ends of the chain hydroxyl groups linked to silicon through an ethyleneoxypropylene moiety. These groups are more reactive than the silanol groups present in classic polysiloxanes. The composition of the prepared urethane acrylates in this study is shown in Table 1. The table shows the optimized composition of the urethane acrylate compositions. The molar ratio of glycerin:xylitol was optimized experimentally. The amount of xylitol used was limited by its limited solubility in glycerol and the viscosity of the reaction mixture. The amount of these components had a significant effect on increasing the crosslinking density of the coatings, but the use of more xylitol caused problems with its miscibility with other components of the reaction mixture and deterioration of the mechanical properties of the coatings. In this manuscript, the largest possible amount of xylitol was used, with which the mechanical properties of the coatings are the best.

**Figure 1:**
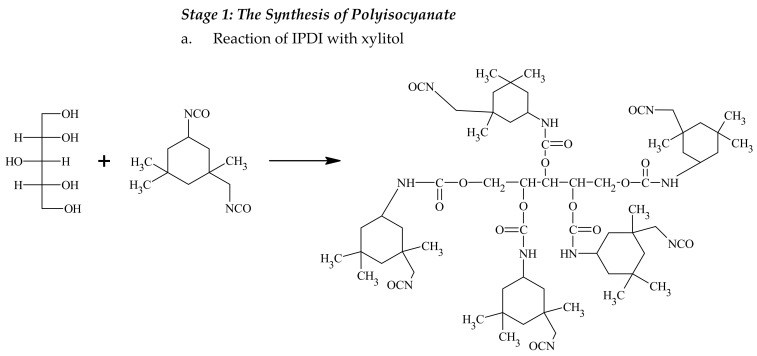

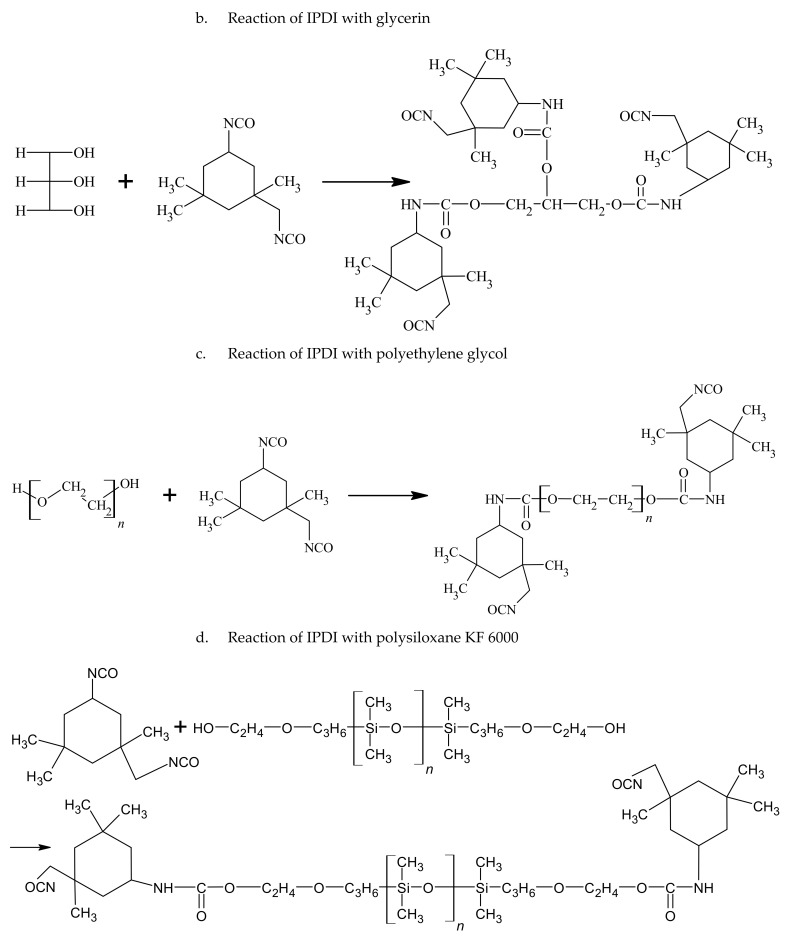

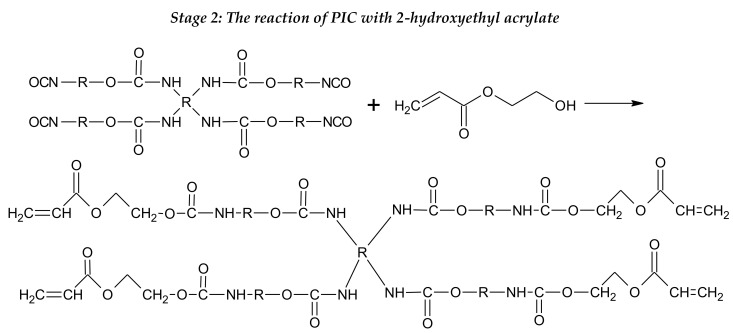
The reaction scheme of the synthesis of urethane acrylates, where -R is mixture of polyisocyanate (**a**–**d**) obtained in the first step of this synthesis.

The synthesis of urethane acrylates included two steps (Figure 1). In the first step, a double excess of -NCO groups derived from IPDI over -OH groups derived from a mixture of glycerin, xylitol, propylene glycol or polysiloxane was used. The use of DBTL catalyst enabled the hydroxyl groups to attach to the secondary cycloaliphatic -NCO groups. Literature data show that the secondary cycloaliphatic isocyanate groups have a higher reactivity when DBTL catalyst is used. The primary isocyanatomethyl group, due to its neighborhood with a methyl group, the cyclohexane ring and β-substituted methyl substituent is effectively covered [30,31,32,33,34,35,36,37]. As the synthesis continued, the viscosity of the mixture increased, which was related to the reaction of the -NCO groups with the -OH groups. The progress of the reaction during the synthesis was examined by the determination of isocyanate groups using the acidimetric method according to PN-EN 1242:2013-06 at 20 min intervals. The first step of the reaction was performed until half of the isocyanate groups (19–20%) had reacted. In this step, a branched PIC with higher functionality was synthesized. In the second step, a compound containing unsaturated bonds was attached to the obtained PIC. 2-hydroxyethyl acrylate or 2-hydroxyethyl methacrylate were chosen as compounds containing the -OH group. The hydroxyl groups, derived from acrylate and methacrylate, can easily react with the -NCO groups and form a stable urethane bond at room temperature. Acrylation and methacrylation reactions are often used in UV-curable systems [6,30]. The amount of 2-hydroxyethyl acrylate or 2-hydroxyethyl methacrylate for the following reaction was computed in relation to the percentage content of -NCO groups. The molar ratio of hydroxyl to isocyanate groups was 1:1. In this step, it was essential to decrease and control the temperature to avoid premature polymerization of unsaturated bonds. The obtained urethane acrylates were transparent, solid and they could be easily powdered, which made it possible to use them as curing agents in UV-curable powder clear coatings.

**Figure 2:**
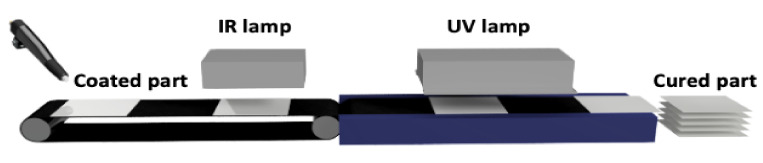
Diagram of technological line for UV-curable powder coatings.

To prepare UV-curable powder clear coatings, an unsaturated polyester resin was used in combination with synthesized urethane acrylates as crosslinking agents and a photoinitiator. Subsequently, to obtain clear coatings with good properties, additives specific to powder clear coatings were applied, including Resiflow PV 88 to improve flowability and benzoin to eliminate gas bubbles. In this study, seven powder coating samples were made. The reference sample contained 100% unsaturated polyester resin and additives. Subsequent samples contained crosslinking agents and unsaturated polyester resin in a mass ratio of 15:85 and additives. During the extrusion process, all coating components were homogenized. The homogenization process was carried out at lower than standard temperatures to avoid premature polymerization of unsaturated bonds at higher temperatures. The new crosslinking agents do not exhibit any detrimental effect on the extrusion and curing processes but due to low Tg of the powder clear coating compositions, the blend should be stored at a temperature below 20 °C. The glass transition temperature of the powder compositions determined by the DMA method were in the range of 75–80 °C, which indicates that under the proposed storage conditions, their particles should not be agglomerated. After obtaining the powder mixture, the next step was curing under UV radiation. A schematic line for this process is shown in Figure 2.

**Figure 3:**
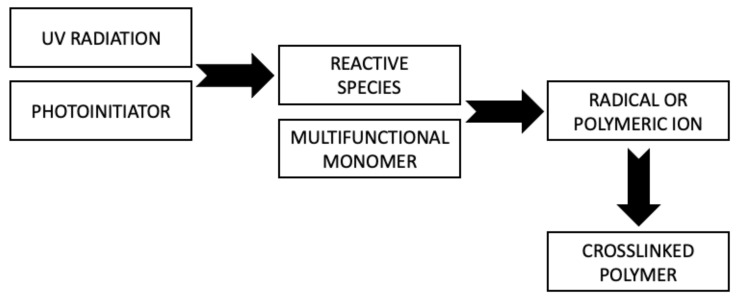
General scheme of photocuring process.

The curing process of the coating occurs through photopolymerization (Figure 3). As a result of a chain reaction initiated by reactive species of photoinitiator generated under UV irradiation, conversion of the multifunctional crosslinking agent into a crosslinked polymer occurs. The use of photoinitiators in this process is essential because most monomers are unable to effectively form reactive molecules under UV radiation [31,32].

**Figure 4:**
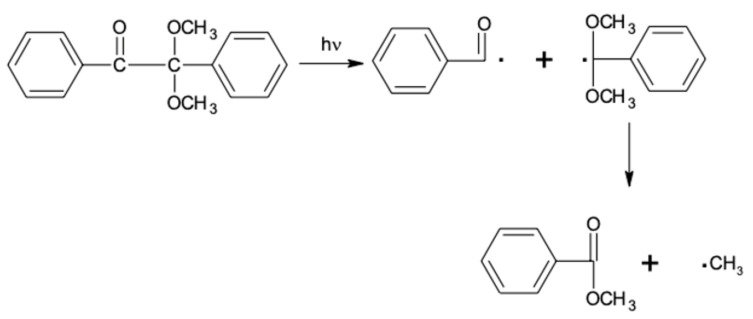
Irgacure 651 radical breakdown reaction.

In our study, the use of unsaturated polyester resin and the obtained unsaturated urethane acrylate as a crosslinking agent combined with Irgacure 651 as a photoinitiator enables the free radical photopolymerization to occur. During the free radical polymerization, the photoinitiator under UV radiation creates free radicals through the homolytic breakdown of the C–C bond, resulting in two different radicals that are able to initiate the polymerization process (type I photoinitiators). The radical breakdown reaction of Irgacure 651 is shown on Figure 4.

**Figure 5:**
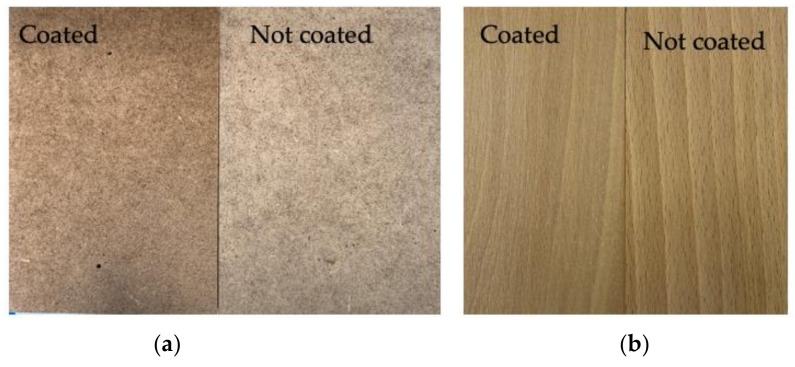
MDF (**a**) and woodplates (**b**) coated using L-IGKFX/HEA.

In the first curing step, the coated steel plates were placed in an oven at 110 °C for 5 min to melt the powder composition components. After that, the plates were placed on a UV line to start the photopolymerization process. A standard mercury lamp with wavelengths <300 and 365 nm was used for the process. To completely cure the powder clear coating, five cycles were performed under a UV lamp. One exposure cycle under the UV lamp lasted 7 s. Temperature-sensitive substrates such as MDF and wood were also used as coating substrates in the study. These substrates were sanded with sandpaper for better adhesion of the coating to their surface. Wood and MDF are also low-conductivity materials that require pretreatment to ensure a conductive surface for electrostatic powder application. Surface activation may be required to improve adhesion. In the case of using wood substrates, it is required to reach a substrate moisture content of 4–8% by infrared preheating to achieve a conductivity that allows the powder to adhere to the substrate during spraying. IR allows for rapid heating of the surface, protecting the core of the board from overheating and preventing cracking of the material. After IR irradiation, the moisture content was measured using a moisture meter. The moisture content for MDF was 6.8% and for wood it was 7.2%. In the next step, the steel, MDF and woodplates were coated using an electrostatic method, and the powder was melted and cured under UV light (Figure 5). The cured coatings were transparent with a uniform surface.

**Figure 6:**
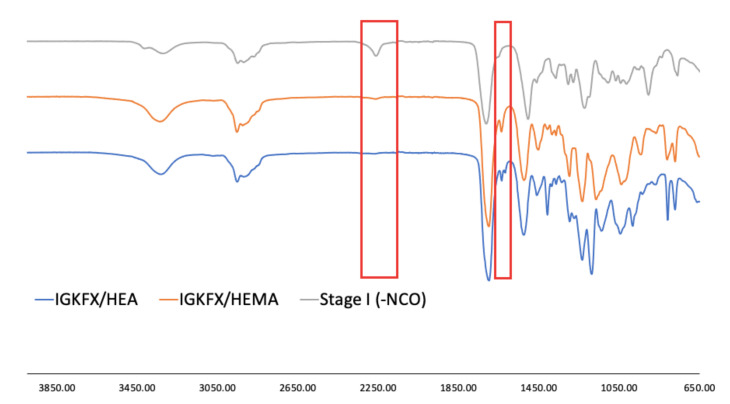
The IR spectra of urethane acrylates IGKFX/HEA and IGKFX/HEMA as well as PIC obtained after stage I of the synthesis.

The chemical structure of the obtained urethane acrylates was confirmed by FT-IR. The interpretation of the spectra is shown in Table 2. The selected spectra are shown in Figure 6. The FT-IR spectra of all obtained products showed no absorption in the range of 2250–2270 cm^−1^, from C-N asymmetric stretching vibration in the -NCO group of diisocyanate. All spectra are essentially identical and show absorption at 3330 cm^−1^ from urethane -NH stretching and at 1521 cm^−1^ from urethane -NH bending vibration as well as stretching vibrations at 1720 cm^−1^ from C=O carbonyl groups. At 1020 cm^−1^ and at 1100 cm^−1^ the Si-O-Si formation of polysiloxane is absorbed, while the polysiloxane Si-CH_3_ vibrations are visible at 1220 cm^−1^ and at 800 cm^−1^. The signal at 810 cm^−1^ is characteristic of unsaturated -C=C-bonds. This signal confirms the incorporation of 2-hydroxyethyl acrylate or 2-hydroxyethyl methacrylate into the PIC structure.

Dynamic mechanical analysis (DMA) was carried out to investigate the curing process of the obtained powder clear coatings. Information about the crosslink density of the cured powder clear coating is derived from the storage modulus E′ in the rubbery plateau. The crosslinking density was calculated using Equation (1):

(1) $$\upsilon =\frac{E_{r}^{\prime }}{3RT_{r}}$$

where:

υ—concentration of elastically effective chains (mol/m^3^),
$E_{r}^{\prime }$—storage modulus at the rubbery plateau (MPa),
R—gas constant (8.314 J/mol K),
$T_{r}$—temperature at which $E_{r}^{\prime }$ was measured.

**Figure 7:**
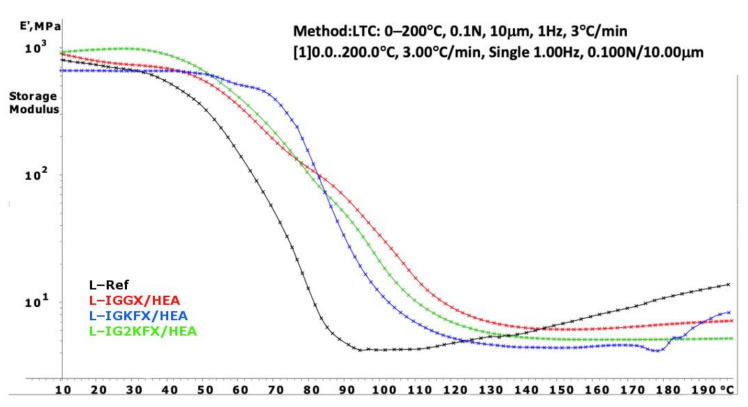
DMA curves of powder clear coatings containing 2-hydroxyethyl acrylate.

**Figure 8:**
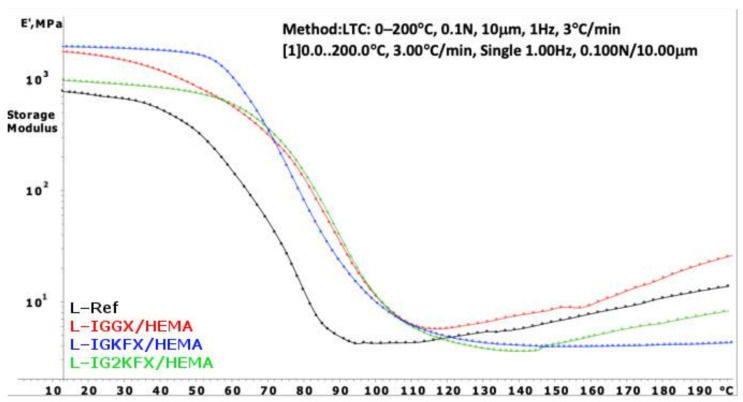
DMA curves of powder clear coatings containing 2-hydroxyethyl methacrylate.

The DMA curves of powder clear coatings are presented in Figure 7 and Figure 8. Using Equation (1), the crosslink density was calculated and is listed in Table 3.

Powder coatings containing HEA have a higher crosslinking density than HEMA-containing coatings. The HEA samples are also characterized with a higher crosslink density compared to the reference sample. From the results, it can be concluded that a denser polymer network was formed when the crosslink density values are higher. The highest crosslink density values were obtained for samples IGGX/HEMA and IGGX/HEA. This is due to the use of 300 g/mol polyethylene glycol in these samples compared to other samples which contain polysiloxane KF-6000 with a molar mass of 1000 g/mol. The longer chains of the polysiloxane made the polymer network less dense. This was also noticeable in the stiffness and brittleness of the coating. The coatings containing polyethylene glycol showed high stiffness and brittleness compared to the coatings containing polysiloxane. The addition of polysiloxane increased the flexibility of the coatings.

**Figure 9:**
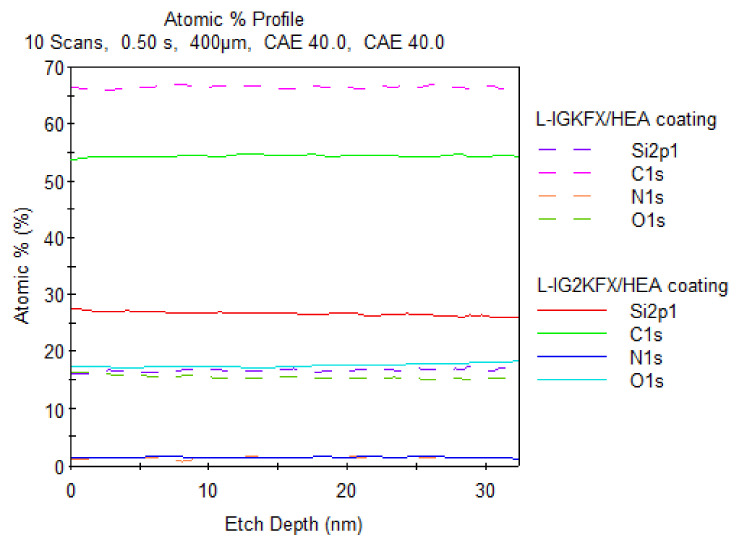
Relationship between the depth of the analysis and the concentration of specific atoms for L-IGKFX/HEA coating and L-IG2KFX/HEA coating.

The chemical composition of the coating surface was investigated using X-ray photoelectron spectroscopy (XPS). Chemical composition is another important factor that influences the hydrophobicity of the coating. The study confirmed the presence of atoms originating from the polyurethane structure (N, O, C) as well as silicon atoms derived from KF-6000 polysiloxane on the coating surface. The test was conducted on the surface of the coating at a depth of 30 nm. The silicon content of the L-IGKFX/HEA coating in accordance with stoichiometric computations is 0.22% and for L-IG2KFX/HEA it is 0.44%. This is due to the use of double the amount of polysiloxane in the crosslinking agent IG2KFX/HEA compared to IGKFX/HEA. After XPS analysis, the percentage of Si content for the L-IGKFX/HEA coating was 16% (Figure 9). For the L-IG2KFX/HEA sample, the silicon percentage at a 30 nm distance is 25% and increases with decreasing distance from the coating surface, and at the coating surface it is 28% (Figure 9). This finding suggests the migration of the polysiloxane chain segments in the direction of the surface. These findings completely confirmed our previous results on polyurethane powder coatings modified with polysiloxane KF 6000 [33] and polysiloxane-containing silanol groups [34]. The cause of this migration is the higher hydrophobicity of the CH_3_ groups that are present in the polysiloxane, which results in thermodynamic incompatibility with the polyurethane segments and an increase in interfacial energy. The reduction in interfacial energy occurs due to the migration of polysiloxane segments toward the surface. The coating shows more silicone-like characteristics if the concentration of polysiloxane on the surface is higher. However, the inhibition of this migration is due to the presence of covalent urethane bonds at the ends of the polysiloxane chains.

After UV curing of the powder coatings, the properties of the coatings on steel panels were tested. The measured property parameters are included in Table 4. Transparent coatings without defects, such as orange peel, cratering or pinholes, were obtained. The highest gloss values were shown by coatings containing HEMA which also resulted in low roughness values. Coatings containing HEA and the reference coating have a visible roughness which results in lower gloss values. It is important to protect the substrate well from the outside environment and provide good decorative properties, and for this it is important to ensure that the coating adheres to the substrate. On a scale of 0–5 (best to worst), the tested coatings showed an adhesion level in the range of 0 to 1. Only coatings containing polysiloxane had the highest parameter on the adhesion scale. The lower adhesion to the substrate of the reference sample and samples containing polyethylene glycol is a result of their high stiffness and brittleness. The relative hardness of the investigated coatings was very good. The addition of a crosslinking agent to the resin increased the hardness of the coating. The reference coating containing only unsaturated polyester resin had the lowest value of hardness. All coatings exhibited good scratch resistance, but coatings containing urethane acrylate modified with KF-6000 showed higher scratch resistance than polyethylene glycol-based urethane acrylate crosslinked coatings. The value of Si-O bond energy (452 kJ/mol) in the polysiloxane structure is higher than polyethylene glycol molecule bond energy (C-O bond energy: 358 kJ/mol, C-C: 347 kJ/mol) [35]. The higher bond energies increase the resistance to breakage of these bonds, which enhances scratch resistance in formulations that include a polysiloxane. Additionally, coatings containing HEA, which have a higher crosslinking density, have higher scratch resistance than those containing HEMA. In addition, the usage of KF 6000 polysiloxane for the synthesis of crosslinking agents increased the water contact angle of the powder coatings. This is because of the presence of hydrophobic segments of the polysiloxane chain on the surface of the coating, as confirmed by XPS analysis. The increment in the contact angle for water shows an improvement in the water resistance of the coating. Hydrophobic coatings provide better protection of the substrate from moisture and easier removal of contaminants from the surface of the coating, which helps to prolong the lifetime of the object that is being protected.

## 5. Conclusions

This work presents a method for the preparation of urethane acrylates as crosslinking agents for UV-curable hydrophobic powder coatings. By using raw materials of renewable origin such as glycerol and xylitol for this synthesis, a product with adequate branching was obtained which also ensures that coatings with good properties were obtained. This offers the possibility of replacing petroleum-based raw materials. Using the DMA method, the crosslinking density was determined with the obtained crosslinking agents and the effect of acrylate and methacrylate reactivity on this crosslinking was shown. As expected, samples crosslinked with HEA were characterized by higher crosslinking density. XPS analysis confirmed the presence of Si atoms in the coating that migrate closer to the surface and thus provide a hydrophobic character. Transparent coatings with uniform layers were successfully achieved. Furthermore, the obtained coatings were characterized by good properties: high hydrophobicity and scratch resistance. These properties are highly essential in the protection of the material surface from environmental factors such as moisture and mechanical damage. The obtained UV-curable powder coatings can be used to coat temperature-sensitive substrates such as MDF and wood, because the heating is only on their surface and does not damage their internal structure. The development of these powder coatings may also help to extend their use to other types of materials with low thermal resistance, such as plastics and composites. This also does not eliminate the possibility of using them to protect metals, such as steel or aluminum, where so-called high-temperature systems have been used. This will provide economic benefits in the form of lower energy consumption.

## Figures and Tables

**Table 1:** Qualitative/quantitative composition of the urethane acrylates.

Name of PIC	IPDI	Glycerin	KF6000	Propylene Glycol 300	Xylitol	HEMA	HEA	Name of Coating
	(mol)	(mol)	(mol)	(mol)	(mol)	(mol)	(mol)	
IGGX/HEMA	0.23	0.058		0.01	0.0057	0.25		L-IGGX/HEMA
IGKFX/HEMA	0.23	0.058	0.005		0.0057	0.29		L-IGKFX/HEMA
IG2KFX/HEMA	0.23	0.062	0.01		0.0057	0.26		L-IG2KFX/HEMA
IGGX/HEA	0.23	0.058		0.01	0.0057		0.27	L-IGGX/HEA
IGKFX/HEA	0.23	0.058	0.005		0.0057		0.26	L-IGKFX/HEA
IG2KFX/HEA	0.23	0.062	0.01		0.0057		0.28	L-IG2KFX/HEA

**Table 2:** Interpretation of FT-IR spectra.

Type of Functional Group	Wave Number [cm^−1^]
-NCO	2250–2270
-NH stretching	3330
-NH bending	1521
-C=O	1720
=CH_2_	810
Si-O-Si	1020 and 1100
Si-CH_3_	1220 and 800

**Table 3:** The crosslinking density of prepared coatings.

Name of Coating	$E_{r}^{\prime }$	$T_{r}$	υ
	(MPa)	(K)	(mol/m^3^)
Reference	4.06	380.25	428.08
L-IGGX/HEMA	5.56	390.4	570.90
L-IGKFX/HEMA	3.98	417.00	382.66
L-IG2KFX/HEMA	3.96	413.40	384.05
L-IGGX/HEA	6.04	423.80	571.40
L-IGKFX/HEA	4.40	413.55	426.57
L-IG2KFX/HEA	5.06	423.00	479.59

**Table 4:** Specifications of coatings properties.

Name of Coating		REF.	L-IGGX/HEMA	L-IGKFX/HEMA	L-IG2KFX/HEMA	L-IGGX/HEA	L-IGKFX/HEA	L-IG2KFX/HEA
Roughness PN-EN ISO 12085	Ra Rz	1.9 17.75	1.31 5.98	1.72 8.87	0.78 3.89	2.75 16.17	2.00 11.20	2.20 11.60
Gloss at an angle of 60° PN-EN ISO 2813	GU	41.40	57.77	69.53	76.52	34.40	41.30	45.20
Adhesion to the steel surface PN-EN ISO 2409	0—best 5—worst	1	1	0	0	1	0	0
Hardness PN-EN ISO 1522	-	0.56	0.79	0.75	0.73	0.80	0.78	0.77
Scratch resistance PN-EN ISO 1518	g	400	500	500	550	500	700	850
Water contact angle EN 828	deg	87.50	89.53	92.19	95.56	88.50	97.37	99.10

## Data Availability

Data is contained within the article.
